# A rapid method for detecting protein-nucleic acid interactions by protein induced fluorescence enhancement

**DOI:** 10.1038/srep39653

**Published:** 2016-12-23

**Authors:** Sona Valuchova, Jaroslav Fulnecek, Alexander P. Petrov, Konstantinos Tripsianes, Karel Riha

**Affiliations:** 1 CEITEC - Central European Institute of Technology, Masaryk University, 625 00 Brno, Czech Republic

## Abstract

Many fundamental biological processes depend on intricate networks of interactions between proteins and nucleic acids and a quantitative description of these interactions is important for understanding cellular mechanisms governing DNA replication, transcription, or translation. Here we present a versatile method for rapid and quantitative assessment of protein/nucleic acid (NA) interactions. This method is based on protein induced fluorescence enhancement (PIFE), a phenomenon whereby protein binding increases the fluorescence of Cy3-like dyes. PIFE has mainly been used in single molecule studies to detect protein association with DNA or RNA. Here we applied PIFE for steady state quantification of protein/NA interactions by using microwell plate fluorescence readers (mwPIFE). We demonstrate the general applicability of mwPIFE for examining various aspects of protein/DNA interactions with examples from the restriction enzyme *BamH*I, and the DNA repair complexes Ku and XPF/ERCC1. These include determination of sequence and structure binding specificities, dissociation constants, detection of weak interactions, and the ability of a protein to translocate along DNA. mwPIFE represents an easy and high throughput method that does not require protein labeling and can be applied to a wide range of applications involving protein/NA interactions.

Interactions of proteins with nucleic acids are crucial for a number of fundamental biological processes including DNA replication and repair, transcription, RNA processing, and translation. For example, binding affinities of transcription factors to promoter and enhancer elements are important determinants of gene expression and a quantitative description of these parameters is crucial for understanding the regulatory networks controlling developmental and physiological processes^1^,^2^. The strength and quality of protein/NA interactions are determined by atomic contacts between amino acids and nucleotides underpinned by physical forces including electrostatic and hydrophobic interactions, hydrogen bonds, and van de Waals forces^3^. Protein/NA interactions are further affected by neighboring proteins, small molecules, and the physical properties of the environment such as temperature or pH. These factors can modulate protein/NA affinities within cells, providing a means for the regulation of biological mechanisms that rely on protein/NA interactions. Therefore, the quantitative description of these interactions under a number of experimental conditions is essential for understanding both physiological processes and human pathologies, and are instrumental for smart drug design^4^.

A wide variety of methods have been developed to describe interactions between proteins and nucleic acids both *in vivo* and *in vitro*. These include chromatin immunoprecipitation, yeast one hybrid assay, microplate capture and ELISA based detection assays, filter binding assay, electrophoretic mobility shift assay (EMSA), fluorescence anisotropy, surface plasmon resonance, kinetic assays based on stopped-flow systems, and single molecule techniques^5^. However, each method has its limitations and a combination of several techniques are often applied for the analysis of a particular interaction. For example, EMSA is one of the most commonly used methods for determining protein/NA affinity and sequence specificity, but it is a rather laborious and time consuming technique with relatively limited throughput that may be insensitive to low-affinity interactions.

For screening even a moderate number of experimental parameters or chemical libraries for drug design, high throughput methods are required. These methods usually use fluorescence that allows rapid detection by fluorescence scanners. Fluorescence anisotropy methods are based on the change in fluorescence polarization upon protein binding to a labeled nucleic acid. In combination with special plate readers such methods can be used in a high throughput format^6^,^7^. Fluorescence anisotropy can provide information about binding affinity and the molecular weight of the resulting complex, but it does not provide positional information. Methods exploiting Förster resonance energy transfer (FRET) that occurs between donor and acceptor fluorophores placed on the protein and nucleic acid are also widely used^8^. These assays allow for a range of experimental settings and can provide spatial information about the interaction. Nevertheless, one disadvantage of FRET-based assays is the requirement for protein labeling, which may be laborious and interfere with the biochemical properties of the proteins being studied.

Here we describe a quantitative, high throughput approach for studying protein/NA interactions without the need for protein labeling. This method exploits protein induced fluorescence enhancement (PIFE), a phenomenon whereby an increase in fluorescence is observed when a protein binds to a fluorescently labeled nucleic acid in the vicinity of a fluorophore^9^. Physical interaction of a protein with cyanine dyes sterically constrains their isomerization, blocking the formation of a fluorescently inactive *cis* isomer^9^. Similar effects have been shown in higher viscosity or lower temperature environments^10^,^11^. PIFE is very specific and has a higher spatial resolution than FRET, showing a sharp response within a 0–3 nm range^12^. The short range and high sensitivity of PIFE proved to be excellent for studying protein/NA interactions in single molecule (smPIFE) experiments^12^,^13^, as well as in kinetic studies using stopped-flow systems^14^,^15^,^16^. We modified PIFE for the quantification of steady-state protein/NA interactions using fluorescent plate readers (microwell PIFE, mwPIFE). We demonstrate the general applicability and versatility of this method in a variety of experiments that probe distinct aspects of protein/NA interactions. These include the association of a protein at different positions along DNA, defining minimal DNA-length binding requirements, determining sequence- or structure-specific DNA binding affinities, and studying the effect of ions on protein binding and nuclease activity. mwPIFE is very simple yet highly quantitative, and can be applied to a broad range of questions involving different aspects of protein/NA interactions.

## Results

### Detection of protein – DNA interaction by mwPIFE

**Figure 1:**
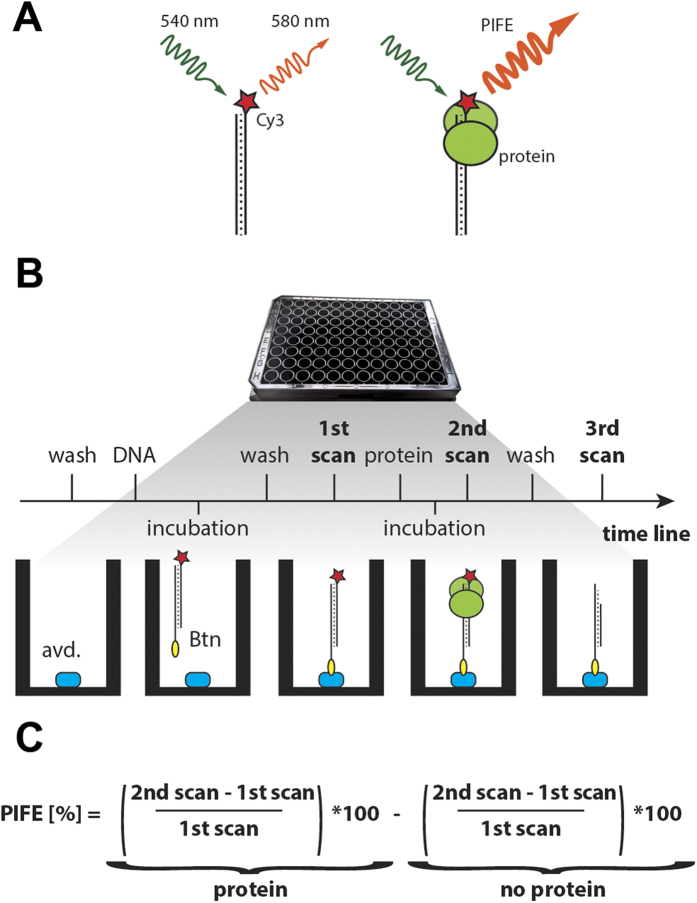
Detection of protein/NA association by mwPIFE. (**A**) Binding of a protein in the physical vicinity of Cy3 leads to fluorescence enhancement. (**B**) Experimental workflow of mwPIFE. Neutravidin coated microwell plate is washed and biotinylated oligonucleotide is bound. The fluorescence of a bound oligonucleotide is measured with a fluorescence plate reader. After incubation with protein a second scan is performed. An optional washing step is followed by a third scan. (**C**) Formula used to calculate PIFE.

In single molecule experiments, the signal emitted from an individual fluorophore can yield a 2- to 2.5-fold PIFE upon protein binding to a Cy3 labeled nucleic acid (Fig. 1A)^12^. We reasoned that the PIFE effect should also be detectable in a population of molecules by standard fluorescence plate readers and would therefore be suitable for more routine protein/NA analyses. We designed an experimental protocol to detect the steady-state interaction of an unlabeled protein with immobilized Cy3-labeled DNA probe by PIFE in a microwell (Fig. 1B). A Cy3-labelled oligonucleotide probe is immobilized to a black microwell coated with neutravidin through biotin attached to either the 5′ or 3′ end of the oligonucleotide. Based on fluorescence measurement of a concentration range of immobilized oligonucleotides we decided to use 2.5 pmol of Cy3- labelled probe (Supplementary Fig. 1). Fluorescence is measured using a plate reader and the signal from the first scan serves as a reference value for determining PIFE. A second scan is performed after incubation of a protein with the DNA probe in the well (Fig. 1B). PIFE is calculated for each well as the relative difference between the signals in the first and second scans. To correct for the effect of buffer and photobleaching on PIFE, a second scan is also performed in a control well where buffer with no protein is added. The final PIFE value represents PIFE in the protein well minus PIFE in the control well (Fig. 1C). A third optional scan can be performed after protein washing in applications that assess protein/DNA dissociation, effect of additional co-factors, or enzymatic activity. PIFE is then calculated relative to the first scan.

**Figure 2:**
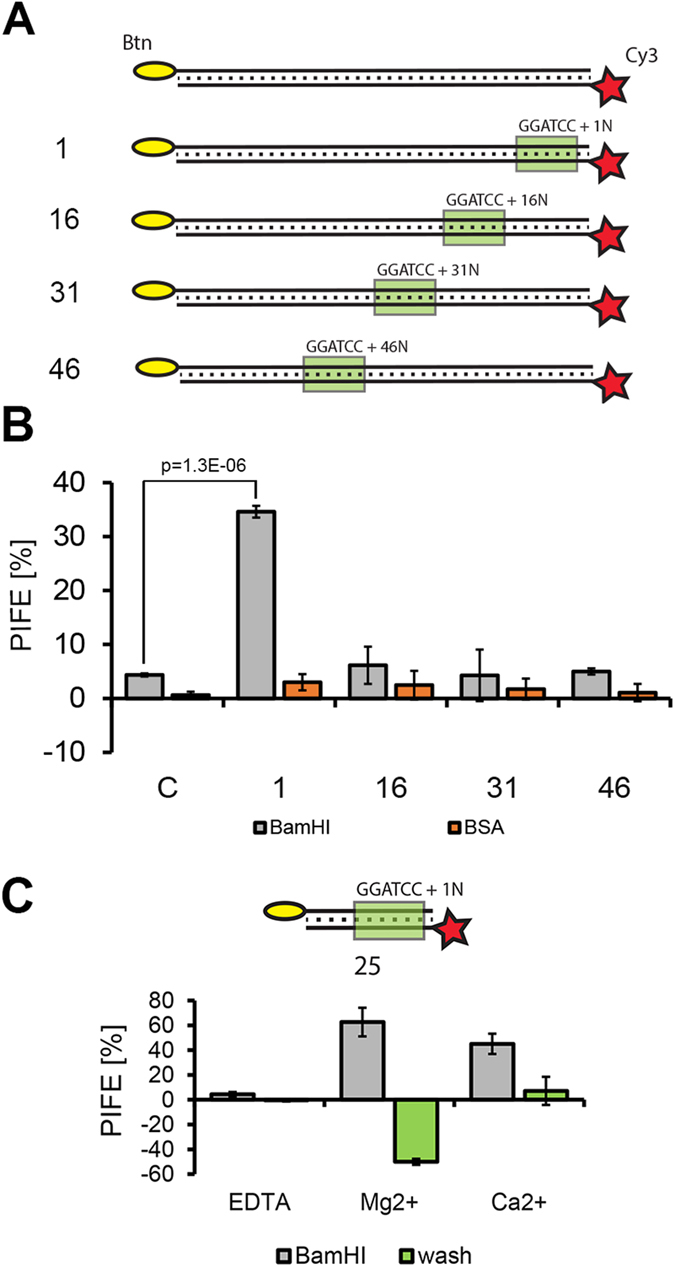
Detection of BamHI/DNA interaction by mwPIFE. (**A**) Scheme of DNA probes for detecting BamHI binding. Position of the BamHI restriction site is indicated. (**B**) Normalized PIFE values obtained with different probes incubated with BSA or BamHI. Error bars indicate standard deviations from measurements of three independent wells. P value was calculated by two-tailed t-test. (**C**) Effect of divalent cations on BamHI binding and cleavage. A 25 bp oligonucleotide with the restriction site 1 bp from Cy3 was used as a probe. Chart shows normalized PIFE values obtained upon addition of BamHI and after the subsequent wash. Error bars indicate standard deviations from measurements of three independent wells.

To test whether the mwPIFE assay used in this format is able to reliably detect protein-DNA association, we examined the interaction of the restriction endonuclease *Bam*HI with its target sequence. A similar experiment was previously performed in a single molecule setup to analyze the distance dependence of PIFE between *Bam*HI and the fluorophore^17^. We prepared 60 bp duplex DNA probes containing *Bam*HI restriction sites either 1, 16, 31, or 46 bp away from Cy3 placed at the 5′ end of one DNA strand, while the opposite strand was attached to microwells through its biotinylated 5′end (Fig. 2A, Supplementary Table 1). A DNA probe of the same length without a *Bam*HI site was used as a control. In the presence of *Bam*HI and Ca^2+^ ions, we detected a 30% PIFE when the binding site was located 1 nucleotide away from the Cy3. Importantly, the PIFE values with *Bam*HI showed a highly significant difference from control reactions with either bovine serum albumin (BSA) or DNA probe with no BamHI site (P values range from 1.2 × 10^−6^ to 1.3 × 10^−6^; two tailed t-test) demonstrating that mwPIFE can reliably detect a protein/NA interaction in a quantitative manner. No PIFE above background was detected with DNA probes where the restriction site was placed 16, 31, or 46 bp away from Cy3 (Fig. 2B), confirming data from the smPIFE study^17^. Thus, similar to the single molecule setup, PIFE detection in a population of molecules is specific for short distance interactions.

### Detection of DNA cleavage and protein dissociation

The mwPIFE setup allows for the testing of a number of experimental conditions and their effect on protein/NA interaction. We demonstrate this by examining the effect of divalent cations on *Bam*HI/DNA binding and cleavage. In the absence of cations, only a weak *Bam*HI induced PIFE was detected. Addition of Mg^2+^ or Ca^2+^ allows efficient binding of *Bam*HI to the restriction site and induced 40–60% PIFE (Fig. 2C). To determine *Bam*HI cleavage activity, a third scan was performed after a 30 min incubation followed by buffer exchange. In the presence of Ca^2+^, mwPIFE was reduced to background level, suggesting that only a small fraction of *Bam*HI remained associated with DNA after washing. The addition of Mg^2+^ ions resulted in a drastic decrease in fluorescence and negative PIFE, which was presumably caused by cleavage and removal of the labeled DNA fragment by washing. These data are consistent with the known properties of *Bam*HI, which has a relaxed requirement of divalent cations for binding, but requires Mg^2+^ for efficient cleavage^18^. This experiment shows that mwPIFE can also be adapted to study protein dissociation and DNA cleavage.

### Assessment of DNA binding specificity

**Figure 3:**
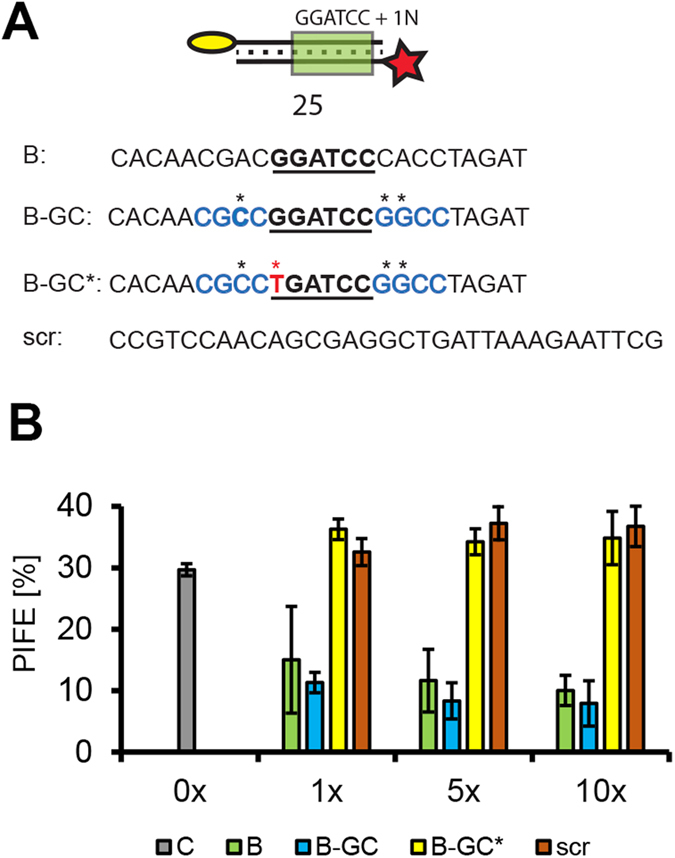
Sequence specificity of BamHI binding. (**A**) The DNA probe and competitors used in the experiment. (**B**) Different dsDNA competitors were added in 1–10x excess over labelled and immobilized DNA probe. PIFE was calculated after incubation with 50U of BamHI. Error bars indicate standard deviations from measurements of three independent wells.

Important aspects of protein/NA interaction are the sequence and structural specificities that can be assessed in competition assays. The high throughout format of mwPIFE is well suited for these experiments as it allows parallel measurements of a number of different competitors in a broad range of concentrations. As a proof of concept, we designed an mwPIFE experiment to test the sequence specificity of *Bam*HI in a competition assay. Binding of *Bam*HI to a Cy3 labeled oligonucleotide containing the restriction site in the presence of Ca^2+^ and four different competitor DNAs was assessed by mwPIFE. When competitor DNA contained substitutions outside of the recognition site (Fig. 3A), it competed with the labeled probe as efficiently as unlabeled probe. However, when a single base was changed within the *Bam*HI site, competition was no longer observed and PIFE was comparable to the reactions where oligonucleotide lacking the BamHI site or no competitor control were added (Fig. 3B). This experiments show that mwPIFE can be used for determining the sequence specificity of a protein/NA interaction.

**Figure 4:**
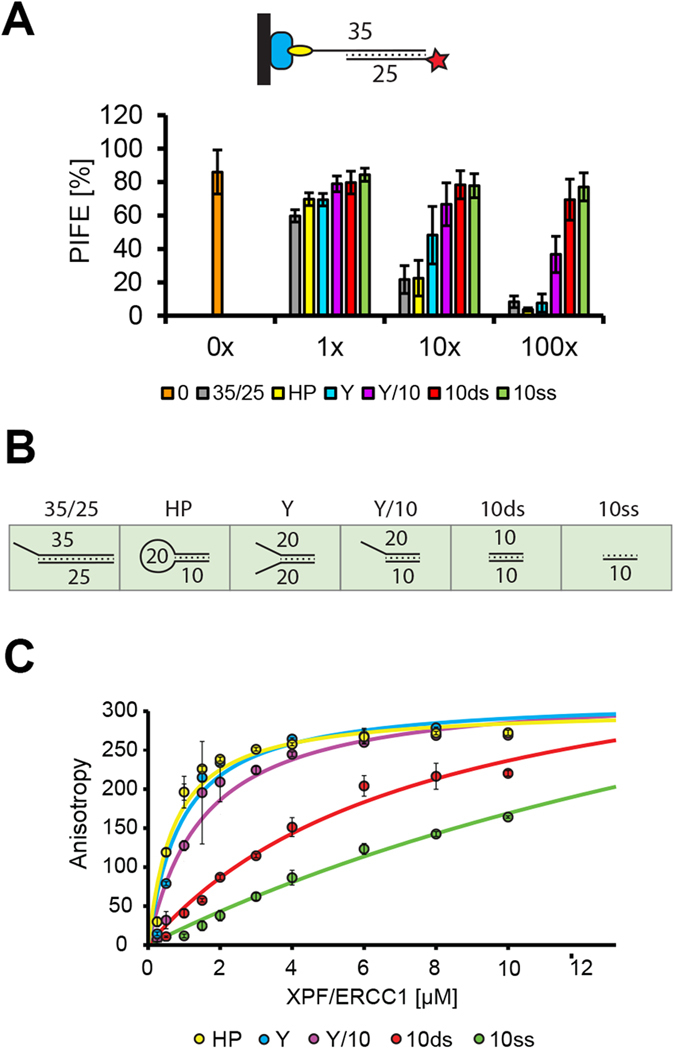
Structure specific binding of XPF/ERCC1. (**A**) Immobilized 35/25 bp DNA with terminal 5′ Cy3 was used as a probe. Different competitors were added in 1x, 10x, and 100x excess and PIFE values were calculated. Error bars indicate standard deviations from measurements of three independent wells. (**B**) Structure of different oligonucleotides used as competitors. Numbers indicate lengths of annealed oligonucleotides in nt. (**C**) Binding curves of XPF/ERCC1 to oligonucleotides used as competitors in panel (**A**) determined by fluorescence anisotropy. Each curve was constructed from 3 independent measurements. Calculated Kd values for each substrate are: HP (0.64 ± 0.1 μM), Y (0.81 ± 0.1 μM), Y/10 (1.32 ± 0.2 μM), 10ds (4.75 ± 0.8 μM), 10ss (8.65 ± 1.3 μM).

Many DNA processing proteins recognize their substrates via specific structures in a sequence independent manner. One of them is the structure-selective endonuclease XPF/ERCC1 that is involved in a number of DNA repair mechanisms including nucleotide excision repair, DNA double strand break repair, and interstrand crosslink repair^19^. XPF and ERCC1 form an obligate heterodimer^20^ that binds and processes branched DNA structures with double-to-single stranded transitions, such as bubbles, stem loops, splayed arms, or overhangs^21^,^22^. To test whether mwPIFE can be used to determine the specificity of structure-dependent interactions, we assessed the association of the XPF/ERCC1 heterodimer with a labeled DNA substrate in the presence of different DNA structures as competitors. Because reconstitution of the full length XPF/ERCC1 in a heterologous expression system is inefficient^23^, we used a truncated heterodimer lacking the N-terminal helicase-like domain of XPF which retains DNA binding and residual cleavage activity^22^,^24^. The mwPIFE binding substrate consisted of a 25 bp duplex DNA with a 10 nt long 3′ overhang (Supplementary Table 1). The DNA probe was immobilized through the end of the single stranded overhang, while the Cy3 label was at the end of the duplex region (Fig. 4A). We observed 80% PIFE in the presence of XPF/ERCC1, indicating efficient binding of the complex to the DNA substrate. Next, we performed the mwPIFE assay whereby the XPF/ERCC1 dimer was added in the presence of increasing concentrations of DNA competitors that included stem-loop, 3′overhang (Y/10), splayed DNA (Y), ssDNA (10ss), and dsDNA (10ds) structures (Fig. 4B). These different competitors exhibited various effect on PIFE. While the stem-loop and splayed DNA exhibited the strongest inhibition of PIFE at higher concentrations, the 3′ overhang showed an intermediate effect and no inhibition was detected for the short ss and dsDNAs (Fig. 4A). We validated this data by determining the dissociation constants (Kd) for each competitor by fluorescence anisotropy (Fig. 4C). Kd values of the competitors are in agreement with their ability to compete for XPF/ERCC1 binding in the mwPIFE assay, demonstrating the utility of mwPIFE in analyzing DNA-structure specific interactions.

### Analysis of Ku-DNA interaction by mwPIFE

**Figure 5:**
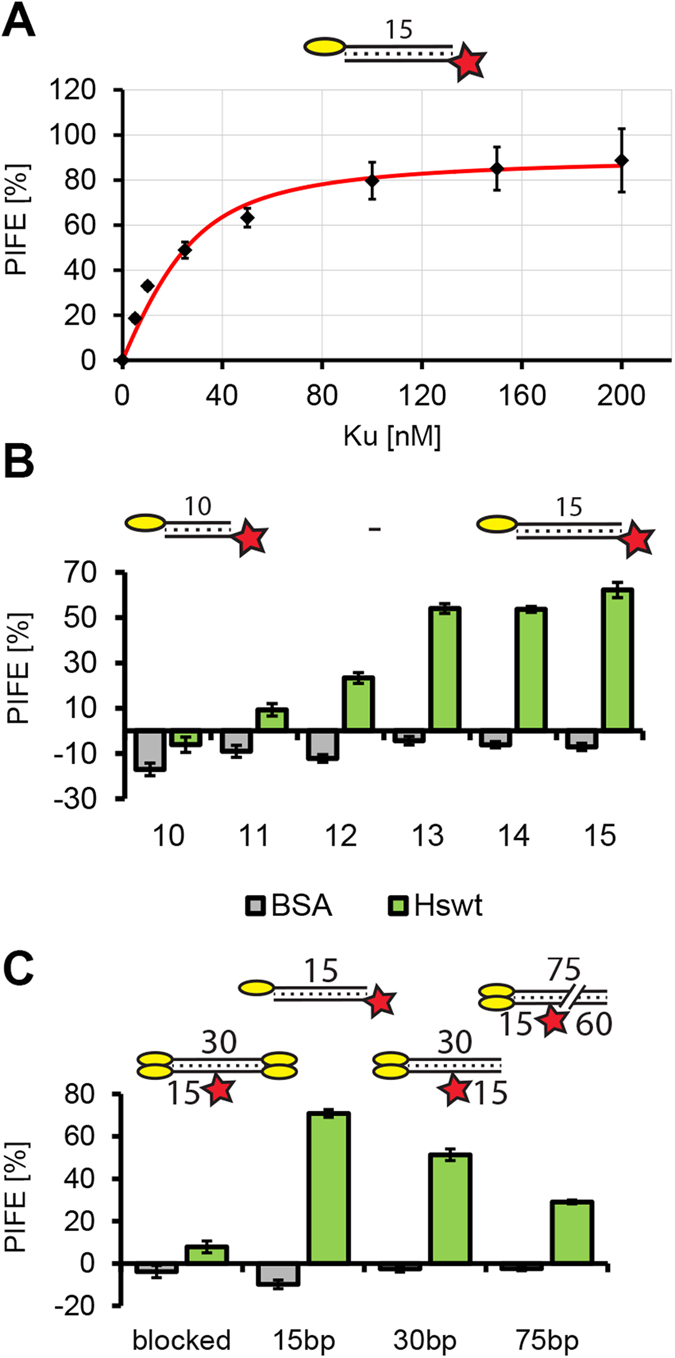
Analysis of Ku-DNA interaction by mwPIFE. (**A**) The magnitude of mwPIFE depends on Ku concentration. A 15 bp duplex oligonucleotide with Cy3 at the free 5′end was used as a probe. The binding curve was reconstituted from 3 independent replicas with standard deviations indicated. (**B**) Minimal binding site of human Ku determined by mwPIFE with 10–15 bp oligonucleotides with 3′ terminal Cy3. Error bars indicate standard deviations from measurements of three independent wells. (**C**) Translocation of Ku estimated by mwPIFE using oligonucleotides with Cy3 at different positions from the DNA termini. Probes used in the experiment are indicated above the chart; numbers indicate length in nucleotides; Cy3 is shown as an asterisk. Normalized PIFE values represent averages form thee replicates. Standard deviations are indicated.

We next analyzed DNA interactions of the human Ku complex, a DNA repair factor with high affinity to DNA ends. Ku is a heterodimer consisting of Ku70 and Ku80 subunits, forming a ring-like structure that loads onto DNA through a free end^25^. Ku-DNA interaction is sequence independent and EMSA has shown it requires at least 14 bp of duplex DNA^26^. We linked a linear 15 bp long dsDNA probe at one end to a microtiter well, while the opposite Cy3 labeled end was available for Ku binding. mwPIFE increased with protein concentration, resulting in a binding curve that reached a plateau around 80% at the highest Ku concentrations (Fig. 5A). The calculated Kd for Ku/DNA interaction using mwPIFE was 10 ± 5 nM, which is within the range previously determined by fluorescence anisotropy experiments^27^.

Next we used mwPIFE to determine the minimum DNA length requirements for Ku/DNA interaction. We measured mwPIFE with terminally labeled dsDNA probes ranging from 10 to 15 bps. An ~50% PIFE was detected with 13, 14, and 15 bp probes, indicating that 13 bp of duplex DNA is sufficient for stable Ku binding. Nonetheless, we also detected a small (7%), but statistically significant PIFE (P = 8.76E-3, two tailed t-test) with an 11 bp probe and a 20% PIFE with the 12 bp probe (Fig. 5B). These signals reflect less stably bound Ku with DNA termini, indicating that mwPIFE can reliably quantify relatively weak and transient protein/NA interactions that may be difficult to detect by other methods.

One of the advantages of PIFE is its dependence on the physical proximity of the protein and fluorescent dye, allowing for the spatial mapping of protein-DNA interactions. Although Ku is known primarily as a DNA end binding protein, it can translocate along naked DNA and reach internal positions. To assess Ku/DNA translocation, we used mwPIFE to measure Ku occupancy at different positions along DNA. We designed duplex DNA probes with Cy3 placed either terminally, or internally 15 or 60 bp away from the free end. We also used an internally labeled probe with both DNA termini blocked by neutravidin. While the strongest PIFE was observed when Cy3 was placed terminally, a specific interaction was still detected at internal sites, albeit with PIFE decreasing with the distance from the free end (Fig. 5C). Only negligible PIFE was detected on the biotin-blocked DNA probe, confirming that Ku requires a free end for efficient binding. This experiment demonstrates that mwPIFE can be used to study the topology of protein/NA binding.

## Discussion

Since the first observations that increasing the viscosity of the local environment increases the fluorescence quantum yield of cyanine dyes, and that this phenomenon can be used to study protein/NA interactions^10^,^28^,^29^, PIFE has gained in popularity particularly in single molecule studies. smPIFE has two major advantages over other fluorescence techniques. First, it does not require protein labeling, which can be laborious and interfere with protein function. Second, it allows for the detection of weaker protein/NA interactions, which would otherwise require high concentrations of fluorescently labeled proteins obscuring single molecule detection^12^. smPIFE has been used in a number of applications including the determination of dissociation and association rate constants, polymerase conformation changes upon nucleotide binding, changes in DNA conformation upon protein binding, helicase translocation along dsRNA, and transcription initiation^13^,^29^,^30^,^31^. These examples show the great versatility of the method in elucidating various biochemical processes involving protein/NA interactions.

Nevertheless, as with other single molecule approaches, smPIFE is a rather advanced technique relying on expensive and specifically tailored instrumentation operated by skilled specialists. In this report, we demonstrate utility of the PIFE phenomenon in detecting steady-state protein/NA interactions in microwell plates using standard fluorescence plate readers. This is a relatively simple assay that requires neither specialized equipment nor extensive training. Bulk measurement of PIFE in a microwell provides an immediate numerical readout of the protein/NA interaction which, together with the possibility of the simultaneous analysis of tens to hundreds of samples, renders this technique highly quantitative. We obtained 40–80% PIFE with three different protein-DNA binding systems, which permitted reliable detection of protein-DNA interactions. The lower mwPIFE values comparing to smPIFE, which can reach up to 250%^12^, are expected as mwPIFE averages signal from large population of labeled oligonucleotides whereby not all of them are bound with a protein. We demonstrate that mwPIFE can be used to determine dissociation constants and to rapidly screen for substrate specificities in competition experiments. The multiple-well plate format of the assay allows for testing a broad range of competitors over a wide range of concentrations, which surpasses the sample processing efficiency of other conventional techniques currently used for this purpose, such as EMSA or fluorescence anisotropy. Data on the affinity of XPF/ERCC1 to different DNA substrates showed that the results obtained by mwPIFE are comparable to those from fluorescence anisotropy, but mwPIFE is more time and cost efficient. Hence, mwPIFE may be particularly useful in high-throughput applications such as searching for optimal binding sites of transcription factors or screening chemical libraries for small molecules that inhibit a particular protein/NA interaction.

smPIFE proved to be an excellent technique for detecting transient and weak interactions, with potential to reveal reaction intermediates^31^,^32^. By analyzing Ku’s affinity to short end-labeled DNA fragments with mwPIFE, we were able to record weak but significant signals even with 11 bp duplexes. The signal became more pronounced with 12 bp DNA, and reached a plateau with 13–15 bp DNA substrates. A similar experiment performed by EMSA determined that the shortest DNA capable of forming a detectable complex with Ku is 14 bp^26^. We suggest that the signal detected by mwPIFE with the 11 and 12 bp probes represents less stable Ku-DNA interactions where DNA does not span the entire Ku-DNA loading channel. This is consistent with photo-crosslinking and crystal structure studies showing that the lagging part of the Ku-DNA loading channel is in direct contact with bases at positions 11 and 13^25^,^26^. Hence, mwPIFE seems to be superior to EMSA in detecting and quantifying weak protein/NA interactions.

A unique feature of PIFE is its dependence on protein binding in the immediate vicinity of the dye. An smPIFE study with three different proteins, including BamHI, established the high sensitivity of the method within a 3 nm range from Cy3^17^. Although we have not performed a fine-tuned distance calibration of mwPIFE, our observation of a marked decrease of PIFE when the binding site of BamHI was moved 15 bp from the dye indicates a similar distance dependence as in the smPIFE setting. This property of PIFE was exploited to study the translocation of proteins along nucleic acids. Typically in these experiments a dye is placed at the DNA termini and the directionality and velocity of the movement was extrapolated from changes in fluorescence over time measured either in smPIFE or stopped-flow settings^13^,^16^,^28^. Here we used mwPIFE to assess the ability of Ku to translocate along duplex DNA by measuring the fluorescence intensity of Cy3 placed at different distances from the DNA end. Since Ku can load onto duplex DNA exclusively from a free DNA end, PIFE at internal positions should reflect Ku’s ability to move along DNA. Similar mwPIFE setups, where Cy3 is placed at different positions along a nucleic acid substrate, can be used to map the topology of protein/NA interactions or formation of higher order structures.

Although the PIFE phenomenon has so far only been used in a relatively limited number of studies, it offers a number of advantages over other assays for protein/NA interactions. Its requirement for standard laboratory equipment, relative ease of the methodological procedure, high throughput format, direct quantitative readout, and wide range of experimental applications are the major advantages that poise mwPIFE to become a wide-spread technique complementing, and perhaps in some cases substituting, other methods that are currently being used.

## Materials and Methods

### Protein preparation

*BamH*I 10U/μl was purchased from Thermo Scientific (catalog number: ER0051). Human Ku heterodimer was reconstituted by co-expression of Ku70 and Ku80 subunits in HI5 insect cells. The Ku80 cDNA was PCR amplified from a HeLa cDNA library (kindly provided by Johannes Popov) using primers Hs80_HL/HR and cloned into BamHI/NotI sites of pFastBac HTA (Invitrogen). The Ku80 cDNA was then reamplified from pFastBac HTA vector using primers Hs80_DL/DR and cloned into XhoI/NheI sites of pFastBac Dual. The Ku70 cDNA was amplified from the HeLa cDNA library using primers Hs70_DL/DR and cloned into BamHI/SpeI sites of pFastBac Dual harboring Ku80. The resulting pFastBac Dual vector was transposed to EMBacY bacmid in *E. coli* and subsequently transfected to Sf9 cells for virus formation. Hi5 insect cells were infected with the virus and harvested 3 days past arrest. Cells were resuspended in lysis buffer (50 mM Tris-Cl, 250 mM KCl, 10% v/v glycerol, 1 mM DTT, pH 8.0) supplemented with protease inhibitors (Roche) and frozen in liquid nitrogen. Thawed cells were spun down and the Ku complex was bound to His Mag Sepharose Ni (GE health care). Beads were washed in lysis buffer containing 50 mM imidazole and Ku was eluted in lysis buffer with 250 mM imidazole. Proteins were filtered using Nanosep centrifugal columns (Pall) and stored at 4 °C.

The human XPF/ERCC1 complex was produced in *Escherichia coli* strain BL21 (DE3) by co-expression of two plasmid vectors. ERCC1 (residues 93–297) was expressed from a modified pET-24d vector and had a dual GST, His-6 tag at the N-terminus with a TEV cleavage site. XPF (residues 640–916) was expressed from pCDF vector without a tag. The recombinant protein heterodimer was extracted from harvested *E. coli* cells in 25 mM Tris-HCl buffer pH 8, 500 mM NaCl, 10 mM Imidazole, 0.01% (v/v) NP40, 10% (v/v) glycerol, 2 mM beta-mercaptoethanol and protease inhibitors. The resulting lysate was applied to a HiTrap IMAC HP column charged with CoCl_2_ according to manufacturer’s instructions (GE Healthcare) and step eluted in buffer supplemented with 500 mM imidazole. The protein containing fractions were incubated at 4 °C with 0.5 mg of TEV protease per 1 L culture for 2 hours and then dialyzed overnight at 4 °C against 25 mM Tris-HCl buffer pH 8, 500 mM NaCl, 10 mM Imidazole, 10% (v/v) glycerol and 2 mM beta-mercaptoethanol. The dialyzed protein sample was passed through the same HiTrap IMAC HP column and the non-bound protein was collected. The protein sample was again dialyzed overnight at 4 °C against 25 mM Tris-HCl pH 7.5, 15 mM NaCl, 10% (v/v) glycerol and 2 mM beta-mercaptoethanol. The dialyzed protein was applied to a Mono Q 4.6/100 PE column (GE HealthCare) and gradient eluted with 10 column volumes of buffer supplemented with 1 M NaCl. The protein containing fractions were combined and applied to a HiLoad 16/600 Superdex 75 pg gel filtration column (GE HealthCare) equilibrated with 25 mM Tris-HCl buffer pH 7.5, 50 mM NaCl and 1 mM TCEP.

### mwPIFE

A black 96 microwell plate coated with 100 μl of NeutrAvidin protein and blocked with 200 μl of SuperBlock blocking buffer (Thermo Scientific, Prod. # 15117) was washed three times with a buffer that was specific for each protein. An oligonucleotide DNA probe of choice (Supplementary Table 1) was annealed beforehand and 2.5 pmol was applied in 100 μl of buffer and incubated for 2 hours in the well in dark. Wells were washed afterwards 3 times with 200 μl of buffer. The first scan was obtained after adding 100 μl of a buffer using the FLUOstar Omega scanner (BMG Labtech) in a well scanning mode with a scan matrix of 10 by 10 points within 6 mm diameter and 10 flashes per scan point. The electrical power in the flash lamp is 0,065 W per flash. Ex540-10 and Em580-10 filters were used for fluorescence measurements of Cy3 labeled probes. After the scan, the buffer was replaced with 100 ul of a buffer containing appropriate protein BamHI (50 U), BSA (15 pmol, Biorad), Ku (0.5–20 pmol) or XPF/ERCC1 (25 pmol). The following binding buffers were used: BamHI buffer (10 mM Tris-HCl pH 8, 100 mM KCl, 0.02% Triton X-100, 0.1 mg/ml BSA) with the addition of either 5 mM EDTA or MgCl_2_ or CaCl_2_; Ku binding buffer (35 mM Tris-Cl pH 7.9, 5.5% glycerol, 150 mM KCl, 1 mM EDTA, 0.1 mM DTT, 1.5 mM imidazol); and XPF/ERCC1 buffer (10 mM Tris-Cl pH 8, 10 mM NaCl). Optionally, DNA competitors were prepared by hybridization of forward and reverse strand (Supplementary Table 1) and added to the mixture together with the protein. Incubations were carried out at room temperature for 30 minutes to reach binding equilibrium and the second scan was performed. PIFE was calculated to correct for initial signal and no protein control (Fig. 1B and text in the result section). Optionally, wells were washed two times with 200 μl of the appropriate buffer with no protein, and the third scan was performed in 100 μl of the buffer.

### Fluorescence anisotropy

The binding analysis of XPF/ERCC1 was performed in 10 mM Tris-HCl buffer pH 8, 10 mM NaCl, 10% (v/v) glycerol and 1 mM TCEP. Reactions containing 50 nM DNA substrate were incubated for 30 min at 25 °C and subsequently transferred to 384-well microplate and read in a Tecan Microplate Reader Infinite F500 (Tecan group Ltd). The data for each protein concentration was averaged over 5 min intervals to remove instrumental noise and processed by subtracting the anisotropy value obtained from a respective DNA substrate without protein. Equilibrium dissociation constants (Kd) were calculated by fitting the data in OriginPro (OriginLab Corporation) to the following equation: FA = (([D] + [P] + Kd) − (([D] + [P] + Kd)^2^ − (4*[D]*[P]))^1/2^) * (A)/(2 * [D]), where [D] and [P] are concentrations of DNA and protein respectively, and A is the maximum anisotropy value.

## Additional Information

**How to cite this article**: Valuchova, S. *et al*. A rapid method for detecting protein-nucleic acid interactions by protein induced fluorescence enhancement. *Sci. Rep.* **6**, 39653; doi: 10.1038/srep39653 (2016).

**Publisher's note:** Springer Nature remains neutral with regard to jurisdictional claims in published maps and institutional affiliations.

## Supplementary Material

Supplementary Information
