# Supplementary material for: A rapid method for detecting protein-nucleic acid interactions by protein induced fluorescence enhancement
Source: Sci Rep. 2016 Dec 23;6:39653. doi: 10.1038/srep39653 (PMC5180085; doi:10.1038/srep39653)
Supplement: Supplementary Information [file srep39653-s1.pdf]

Supplementary information

A rapid method for detecting protein-nucleic acid interactions by protein induced fluorescence enhancement

Sona Valuchova, Jaroslav Fulnecek, Alexander P. Petrov, Konstantinos Tripsianes, Karel Riha

Supplementary Figure 1

Supplementary table 1

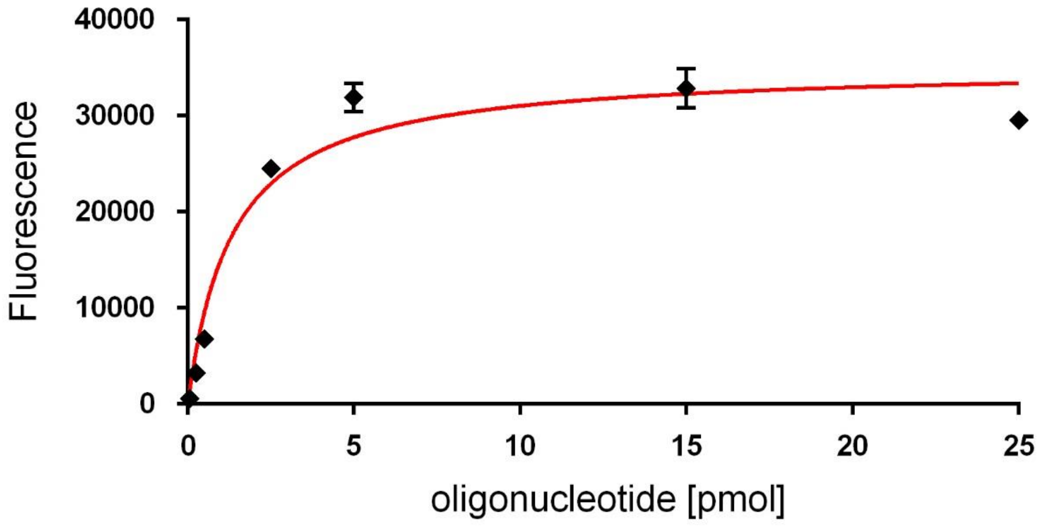

Supplementary Figure 1

The binding curve of 25 bp oligonucleotide Btn_25_f/ Cy3_25_r to a microwell plate. Increasing amount of 25 bp biotinylated oligonucleotide was immobilized to a microwell plate in triplicate. Plotted fluorescence represents the average fluorescence signal of triplicate reduced by signal from a well with a binding buffer to correct for background. Standard deviations are indicated. We consider the range of 0.5 – 5 pmol of oligonucleotide suitable for PIFE measurements. The 2.5 pmol concentration of oligonucleotides was used throughout the study as it sufficient fluorescence without saturating the capacity of microplate wells (binding capacity: > 15pmol D-biotin/well).

Supplementary Table 1. List of oligonucleotides used in the study

Primer name	sequence
Hs80_HL	ATATGGATCCAGTGCGGTCGGGGAATAAGGCA
Hs80_HR	ATATGCGGCCGCTTATATCATGTCCAATAAATC
Hs80_DL	ATATCTCGAGGTCCGAAACCATGTCGTA
Hs80_DR	ATATGCTAGCTTATATCATGTCCAATAA
Hs70_DL	ATATGGATCCATGTCAGGGTGGGAGTCATAT
Hs70_DR	ATATACTAGTTTAGTCCTGGAAGTGCTTGGT
C_f	[Btn]CACAACGACCACCTAGATAGAGCCAAGCAGTTTACGTACCGTCATTCCCTTCCACGTCATCGAGCTACCGTCGTC
C_r	[Cyanine3]GACGACGGTAGCTCGATGACGTGGAAGGGAATGACGGTACGTAAACTGCTTGGCTCTATCTAGGTGGTCGTTGTG
1_f	[Btn]CACAACGACCACCTAGATAGAGCCAAGCAGTTTACGTACCGTCATTCCCTTCCACGTCATCGAGCTACGGATCCA
1_r	[Cyanine3]TGGATCCGTAGCTCGATGACGTGGAAGGGAATGACGGTACGTAAACTGCTTGGCTCTATCTAGGTGGTCGTTGTG
16_f	[Btn]CACAACGACCACCTAGATAGAGCCAAGCAGTTTACGTACCGTCATTCCCTTCCGGATCCACGAGCTACCGTCGTC
16_r	[Cyanine3]GACGACGGTAGCTCGTGGATCCGGAAGGGAATGACGGTACGTAAACTGCTTGGCTCTATCTAGGTGGTCGTTGTG
31_f	[Btn]CACAACGACCACCTAGATAGAGCCAAGCAGTTTACGTAGGATCCTTCCCTTCCACGTCATCGAGCTACCGTCGTC
31_r	[Cyanine3]GACGACGGTAGCTCGATGACGTGGAAGGGAAGGATCCTACGTAAACTGCTTGGCTCTATCTAGGTGGTCGTTGTG
46_f	[Btn]CACAACGACCACCTAGATAGAGCGGATCCGTTTACGTACCGTCATTCCCTTCCACGTCATCGAGCTACCGTCGTC
46_r	[Cyanine3]GACGACGGTAGCTCGATGACGTGGAAGGGAATGACGGTACGTAAACGGATCCGCTCTATCTAGGTGGTCGTTGTG
Btn_25BamHI1_f	[Btn]CACAACGACCACCTAGATGGATCCA
Cy3_25BamHI1_r	[Cy3]TGGATCCATCTAGGTGGTCGTTGTG
B_f	CACAACGACGGATCCCACCTAGAT
B_r	ATCTAGGTGGGATCCGTCGTTGTG
B-GC_f	CACAACGCCGGATCCGGCCTAGAT
B-GC_r	ATCTAGGCCGGATCCGGCGTTGTG
B-GC*_f	CACAACGCCTGATCCGGCCTAGAT
B-GC*_r	ATCTAGGCCGGATCAGGCGTTGTG
scr_f	CCGTCCAACAGCGAgGCTGATTAAAGAATTCG
scr_r	CGAATTCTTTAATCAGCcTCGCTGTTGGACGG
XPF_35_Btn_f	[Btn]TTGCATCCTTCACAACGACCACCTAGATAGAGCCA
XPF_25_Cy3_r	TGGCTCTATCTAGGTGGTCGTTGTG[Cy3]
XPF_35_f	TTGCATCCTTCACAACGACCACCTAGATAGAGCCA
XPF_25_R	TGGCTCTATCTAGGTGGTCGTTGTG
XPF_HP	CAGCGCTCGGTTTTTTTTTTTTTTTTTTTTCCGAGCGCTG
XPF_Yr	TTTTTTTTTTCCGAGCGCTG
XPF_Yf	CAGCGCTCGGTTTTTTTTTT

XPF_10f	CAGCGCTCGG
XPF_10r	CCGAGCGCT
Btn_10_f	[Btn]GATAGAGCCA
Cy3_10_r	[Cy3]TGGCTCTATC
Btn_11_f	[Btn]AGATAGAGCCA
Cy3_11_r	[Cy3]TGGCTCTATCT
Btn_12_f	[Btn]TAGATAGAGCCA
Cy3_12_r	[Cy3]TGGCTCTATCTA
Btn_13_f	[Btn]CTAGATAGAGCCA
Cy3_13_r	[Cy3]TGGCTCTATCTAG
Btn_14_f	[Btn]CCTAGATAGAGCCA
Cy3_14_r	[Cy3]TGGCTCTATCTAGG
Btn_15_f	[Btn]ACCTAGATAGAGCCA
Cy3_15_r	[Cy3]TGGCTCTATCTAGGT
Btn_25_f	[Btn]CACAACGACCACCTAGATAGAGCCA
Cy3_25_r	[Cy3]TGGCTCTATCTAGGTGGTCGTTGTG
BtnT_15_Cy3_15_ BtnT _f	[5BioTinTEG]TT GCA TCC TTC ACA A[iCy3]C GAC CAC CTA GAT AG[3BioTEG]
BtnT_30_ BtnT _r	[5BioTinTEG]CTA TCT AGG TGG TCG TTG TGA AGG ATG CAA [3BioTEG]
BtnT_15_Cy3_15_f	[5BioTinTEG]TT GCA TCC TTC ACA A[iCy3]C GAC CAC CTA GAT AG
30_BtnT_r	CTA TCT AGG TGG TCG TTG TGA AGG ATG CAA [3BioTEG]
BtnT_15_Cy3_60_f	[5BioTinTEG]TT GCA TCC TTC ACA A[iCy3]C GAC CAC CTA GAT AGA GCC AAG CAG TTT ACG TAC CGT CAT TCC CTT CCA CGT CAT CGA GC
75_BtnT_r	GCT CGA TGA CGT GGA AGG GAA TGA CGG TAC GTA AAC TGC TTG GCT CTA TCT AGG TGG TCG TTG TGA AGG ATG CAA [3BioTEG]
